# Novel variants in *COL2A1* causing rare spondyloepiphyseal dysplasia congenita

**DOI:** 10.1002/mgg3.1139

**Published:** 2020-01-23

**Authors:** Wen‐bin Zheng, Lu‐jiao Li, Di‐chen Zhao, Ou Wang, Yan Jiang, Wei‐bo Xia, Xiao‐ping Xing, Mei Li

**Affiliations:** ^1^ Key Laboratory of Endocrinology Department of Endocrinology National Health and Family Planning Commission Peking Union Medical College Hospital Chinese Academy of Medical Sciences and Peking Union Medical College Beijing China

**Keywords:** *COL2A1*, novel variants, spondyloepiphyseal dysplasia congenita

## Abstract

**Background:**

Spondyloepiphyseal dysplasia congenita (SEDC) is an extremely rare inherited chondrodysplasia characterized by abnormal epiphyses, short stature, and flattened vertebral bodies. We investigate the phenotypes and the disease‐associated variants of SEDC in two unrelated Chinese families.

**Methods:**

We identified disease‐associated variants in two nonconsanguineous families with SEDC using targeted next‐generation sequencing and confirmed the variants using Sanger sequencing. We investigated the phenotypes of the patients, including clinical manifestations, bone turnover biomarkers, bone mineral density and skeletal radiographic features.

**Results:**

Two probands were diagnosed as SEDC according to the phenotypes of disproportionately short‐trunk stature, kyphosis, lumbar lordosis and adduction deformity of hips. Radiographs revealed kyphosis and lumbar lordosis, flattened vertebral bodies, compressed femoral heads and shortening of the femurs. Bone mineral density of the probands was lower than that of age‐ and gender‐matched normal children, but bone turnover biomarker levels were within normal range. Two novel heterozygous missense variants (NM_001844.5: c.1654 G>A, NP_001835.3: p.Gly552Arg; NM_001844.5: c.3518G>T, NP_001835.3: p.Gly1173Val) in collagen type II alpha 1 chain (*COL2A1*) were detected in the two families, which would impair the formation of stable triple‐helical type II collagen.

**Conclusions:**

We identified two novel disease‐associated variants in *COL2A1*, which led to severe SEDC. Our findings expanded the gene variant spectrum and phenotypic spectrum of extremely rare type II collagenopathies.

## Graphical abstract

The phenotype and two novel heterozygous missense mutations (NM_001844.5: c.1654 G>A, NP_001835.3: p.Gly552Arg; NM_001844.5: c.3518G>T, NP_001835.3: p.Gly1173Val) in collagen type II alpha 1 chain in the two families were detected. Our study expanded the genetic spectrum and clinical features of spondyloepiphyseal dysplasia congenita.

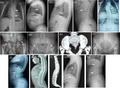

## 1 INTRODUCTION

Spondyloepiphyseal dysplasia congenita (SEDC; OMIM#183900) is an extremely rare autosomal dominant inherited chondrodysplasia, which is characterized by epiphyseal dysplasia in vertebral bodies and long bones (Spranger & Langer, 1970). The prevalence of SEDC is about 3.4 per million (Spranger & Langer, 1970). The phenotypes of SEDC widely vary, which include disproportionately short structure, short neck, skeletal and vertebral deformities, such as scoliosis and thoracic hyperkyphosis, coxa vara, irregular short femoral necks, flat acetabular roofs, genu valgum, and various joint diseases (Liu et al., 2016; Xu, Qiu, Zhu, Yi, & Qiu, 2014). Skeletal abnormalities in SEDC patients usually appear at birth and gradually progress. The extra‐skeletal manifestations include hearing loss, cleft palate and myopia with retinal detachment (Cao et al., 2012; Hamidi‐Toosi & Maumenee, 1982; Ishida, Koh, Kaito, & Nishida, 2018; Xu, Qiu, Zhu, Yi, & Qiu, 2014).

According to the recent genetic investigations, most SEDC patients are caused by variants in collagen type II alpha 1 chain (*COL2A1*), which is located on 12q13.11‐q13.2 and composed of 54 exons. Collagen type II alpha 1 chain encodes α‐1 chain of type II procollagen, which has 1,487 amino acids and contains a triple‐helical domain formed by 330 Gly‐X‐Y triplets (Anderson, Goldberg, Marion, Upholt, & Tsipouras, 1990; Barat‐Houari, Sarrabay, et al., 2016). Type II procollagen is the main structural protein of the cartilaginous tissues, and its changes are involved in many forms of chondrodysplasias (Nishimura et al., 2005). The variants in *COL2A1* often lead to amino acid substitution in the triple helix domain of α‐1 chain of type II procollagen, which affects a glycine required for correct conformation of the triple helix and leads to severe phenotypes (Barat‐Houari, Dumont, et al., 2016; Barat‐Houari, Sarrabay, et al., 2016).

As far as we know, more than 500 different variants in *COL2A1* have been identified and over 50 variants in *COL2A1* are responsible for SEDC, without particular a hot spot variant (BIOBASE Human Gene Mutation Database professional 2019.1, HGMD; http://www.hgmd.cf.ac.uk/ac/index.php; Leiden Open Variation Database 3.0, LOVD 3.0, http://www.lovd.nl/3.0/home). However, different variants in *COL2A1* can result in same or similar phenotypes, which make it difficult to clarify the genotype–phenotype correlations in SEDC. In a study of SEDC, most variants in *COL2A1* were heterozygous missense variants which resulted in a glycine substitution in the triple‐helical domain of the type II procollagen chain, and glycine‐to‐serine substitutions were the most common (Terhal et al., 2015). Other studies indicated that the most frequent variant of p.Arg989Cys in *COL2A1* led to severe skeletal dysplasia, and glycine‐to‐serine substitutions were associated with mild SEDC (Silveira et al., 2015; Steplewski et al., 2004). However, detailed studies about pathogenic gene variants in SEDC are very limited in China (Cao et al., 2012; Chung, Luk, Lo, Lam, & Li, 2013; Huang et al., 2015; Li, Ma, Wang, Cui, & Xiao, 2015; Li, Zhou, Qin, Guo, & Bai, 2014; Liu et al., 2016; Xiong et al., 2018; Xu et al., 2014; Zhang, He, Fu, Zhang, & Zhang, 2011).

In this study, we investigated the phenotypes and the pathogenic variants of two unrelated Chinese families with SEDC in detail, in order to elucidate the molecular mechanism of the rare disorder.

## 2 MATERIALS AND METHODS

### 2.1 Patients

**Figure 1:**
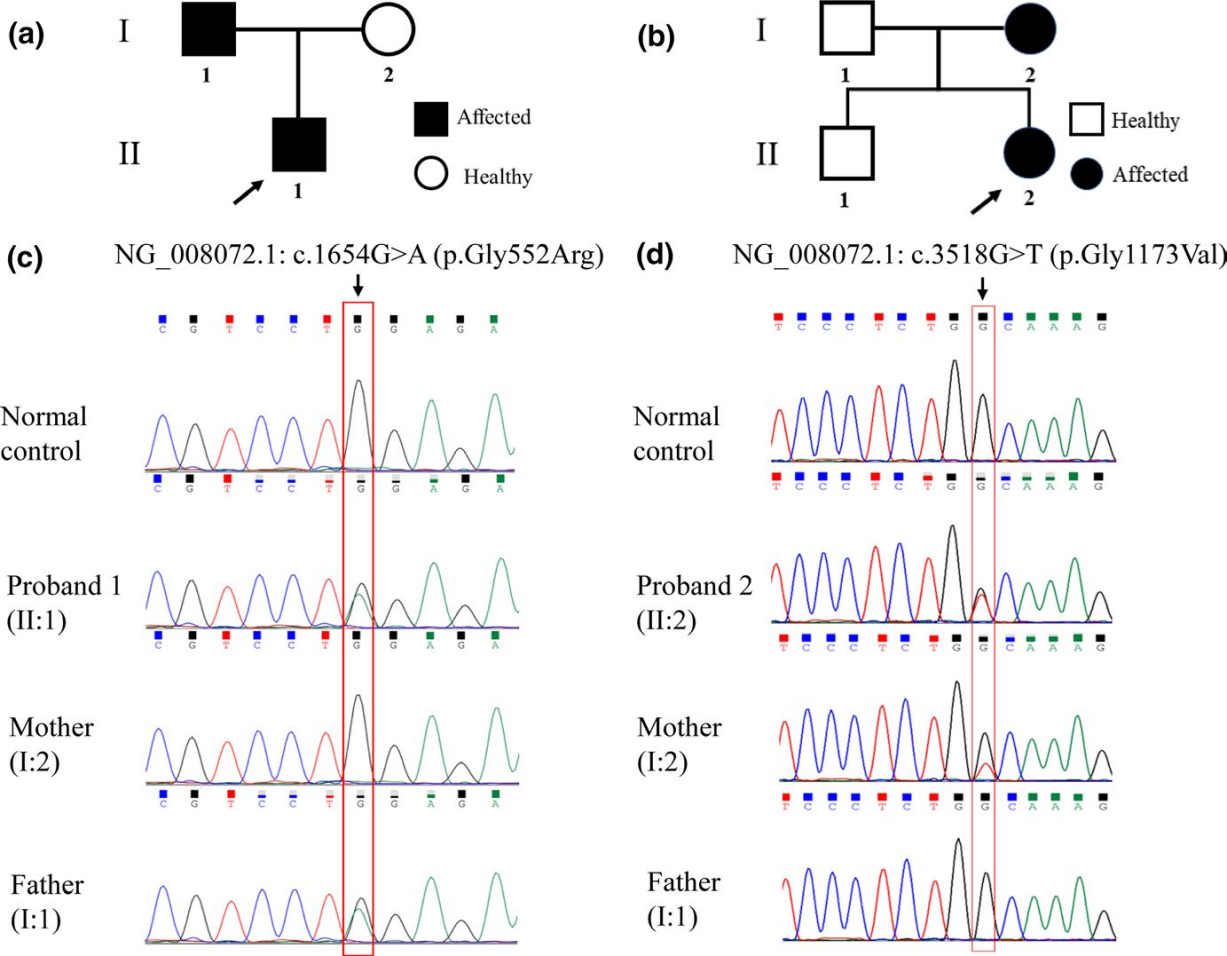
Pedigree of the spondyloepiphyseal dysplasia congenita families and Sanger sequencing results of *COL2A1*. (a, b) The pedigree of the family 1 and 2 in this study. The probands are indicated by black arrow. (c, d) The DNA sequencing results of the patients, their families and normal controls. In proband 1 and his father, a novel variant was identified as c.1654G>A (p.Gly552Arg) in exon 25 of *COL2A1*. In proband 2 and her mother, a novel variant was identified as c.3518G>T (p.Gly1173Val) in exon 50 of *COL2A1*

The probands from two unrelated families of Chinese Han origin were clinically diagnosed as SEDC in Endocrinology Department of Peking Union Medical College Hospital (PUMCH) between 2018 and 2019. The pedigrees of these two families are shown in Figure 1.

Additional unrelated 100 healthy individuals were recruited as normal controls for this study. The study was approved by the Scientific Ethics Committee of PUMCH. Signed informed consents were obtained from the members of the families and the controls before they participated in this study.

### 2.2 Phenotypic evaluation

A detailed medical history was collected and physical examination was completed. Height and weight of patients were measured by Harpenden stadiometer (Seritex Inc., East Rutherford, NJ, USA), and were adjusted to age‐ and gender‐specific *Z*‐scores on the basis of reference data of the Chinese children (Li, Ji, Zong, & Zhang, 2009).

Bone deformities of spine, pelvis and limbs were evaluated using X‐ray films and three‐dimensional (3D) computed tomography (CT) scan. Bone mineral density (BMD) at lumbar spine (LS) 2–4 and proximal hip were detected using dual‐energy X‐ray absorptiometry (Lunar Prodigy Advance; GE Healthcare). Bone mineral density data were transformed to age‐ and gender‐specific *Z*‐scores according to normal reference of BMD in Asian children (Khadilkar, Sanwalka, Chiplonkar, Khadilkar, & Mughal, 2011). Serum levels of beta cross‐linked carboxy‐terminal telopeptide of type I collagen (β‐CTX, a bone resorption marker), 25‐hydroxyvitamin D (25OHD) and intact parathyroid hormone (PTH) were measured using an automated electrochemiluminescence system (Roche Diagnostics). Serum levels of calcium (Ca), phosphorus (P), alkaline phosphatase (ALP, a bone formation marker), aminotransferase, and creatinine (Cr) were measured using an automatic biochemistry analyzer.

### 2.3 Disease‐associated variant identification

Genomic DNA was extracted from peripheral leukocytes under the standard protocol (QIAamp DNA, Qiagen). To identify the sequence variant, a targeted next‐generation sequencing (NGS) panel (Illumina HiSeq2000 platform; Illumina, Inc.) was used to capture all exon sequences of over 700 genes involved in skeletal disorders. Bioinformatic analysis was carried out following the pipelines previously described (Liu et al., 2017). To confirm the gene variant, fragments covering the variant sites in *COL2A1* identified usingb NGS were amplified usingb polymerase chain reaction (PCR) in the probands with SEDC and their parents, as well as 100 healthy controls. Primers were designed using the online Primer 3 (http://bioinfo.ut.ee/primer3-0.4.0/). Primer sequences of *COL2A1* (NM_001844.5) are as follows: exon 25: forward 5′‐GTCAAGATGGTCTGGCAGGT‐3′ and reverse 5′‐GCCAGTGAGACCCTTTGTTC‐3′; exon 50: forward 5′‐GACTGAGCATGTGAAGAACTGG‐3′ and reverse 5′‐TGGTAGGGACACCTCGACAG‐3′. Polymerase chain reaction was conducted with TaqMix DNA polymerase (Biomed) under the standard conditions: denaturation at 95°C for 3 min, followed by 35 cycles at 95°C for 30 s, annealing temperature 60°C for 30 s, and 72°C for 1 min and by a final extension at 72°C for 15 min. The purified PCR products were directly sequenced on the ABI377 DNA automated sequencer using BigDye Terminators Cycle Sequencing Ready Reaction Kit (Applied Biosystems).

Abnormal sequences were identified by comparison with the NCBI reference sequence (GenBank: NG_008072.1) using Chromas 2.6.5. Novel variants were confirmed by comparison with HGMD (http://www.hgmd.cf.ac.uk/ac/index.php), LOVD3.0 (http://www.lovd.nl/3.0/home) and literature of PubMed database. The potential deleterious effects of the detected variant were predicted using PolyPhen‐2 (http://genetics.bwh.harvard.edu/pph2/), SIFT (http://sift.jcvi.org/) and MutationTaster (http://www.mutationtaster.org/). Online UniProt software (http://uniprot.org/) was used to analyze the conservatism of the amino acid substitution among species. Interpretation of variants' pathogenicity was conducted via VarSome link (https://varsome.com/about/acmg-implementation/) according to the American College of Medical Genetics and Genomics (ACMG) guidelines (Richards et al., 2015).

We reviewed previously reported SEDC cases and found eight variants of glycine‐to‐valine substitution in nine patients (Cao et al., 2012; Jung et al., 2004; Li et al., 2014; Liu et al., 2016; Rukavina et al., 2014; Terhal et al., 2015) and seven variants of glycine‐to‐arginine substitution in eight cases (including our patients; Barat‐Houari, Dumont, et al., 2016; Polla et al., 2015; Sobetzko, Eich, Kalff‐Suske, Grzeschik, & Superti‐Furga, 2000; Vikkula et al., 1993). The clinical and variant characters were analyzed to explore the genotype–phenotype correlation.

## 3 RESULTS

### 3.1 Clinical phenotypes

The clinical characters of the patients are presented in Table 1

**Table 1:** Clinical, biochemical markers and BMD of the patients with SEDC

	Family 1		Family 2		Reference range
	II:1	I:1	II:2	I:2	
Age (year)	2	33	11	44	/
Ht (cm)	78	150	110.5	146	/
Wt (kg)	12.5	56	31	58	/
ALT (U/L)	16	NA	12	NA	7–40
Cr (μmol/L)	29	NA	27	NA	18–69
Ca (mmol/L)	2.52	NA	2.56	NA	2.13–2.70
P (mmol/L)	1.66	NA	1.72	NA	1.29–1.94
ALP (U/L)	273	NA	276	NA	42–390
β‐CTX (ng/ml)	2.1	NA	1.31	NA	/
25OHD (ng/ml)	21	NA	24.4	NA	30–60
PTH (pg/ml)	32.3	NA	21.9	NA	12.0–68.0
LS‐BMD (g/cm^2^)	0.329	NA	0.463	1.104	/
LS‐BMD *Z*‐score	−3.4	NA	−2.3	0	/
FN‐BMD (g/cm^2^)	0.486	NA	0.574	1.048	/
FN‐BMD *Z*‐score	−1.6	NA	−2.1	1.7	/

Abbreviations: 25OHD, 25 hydroxyvitamin D; β‐CTX, β‐isomerized carboxy‐telopeptide of type I collagen; ALP, alkaline phosphatase; ALT, alanine aminotransferase; BMD, bone mineral density; Ca, Serum calcium; Cr, creatinine; FN, femoral neck; Ht, height; LS, lumbar spine; NA, not available; P, Serum phosphate; PTH, parathyroid hormone; SEDC, spondyloepiphyseal dysplasia congenita, Wt, weight.

#### 3.1.1 Family 1

**Figure 2:**
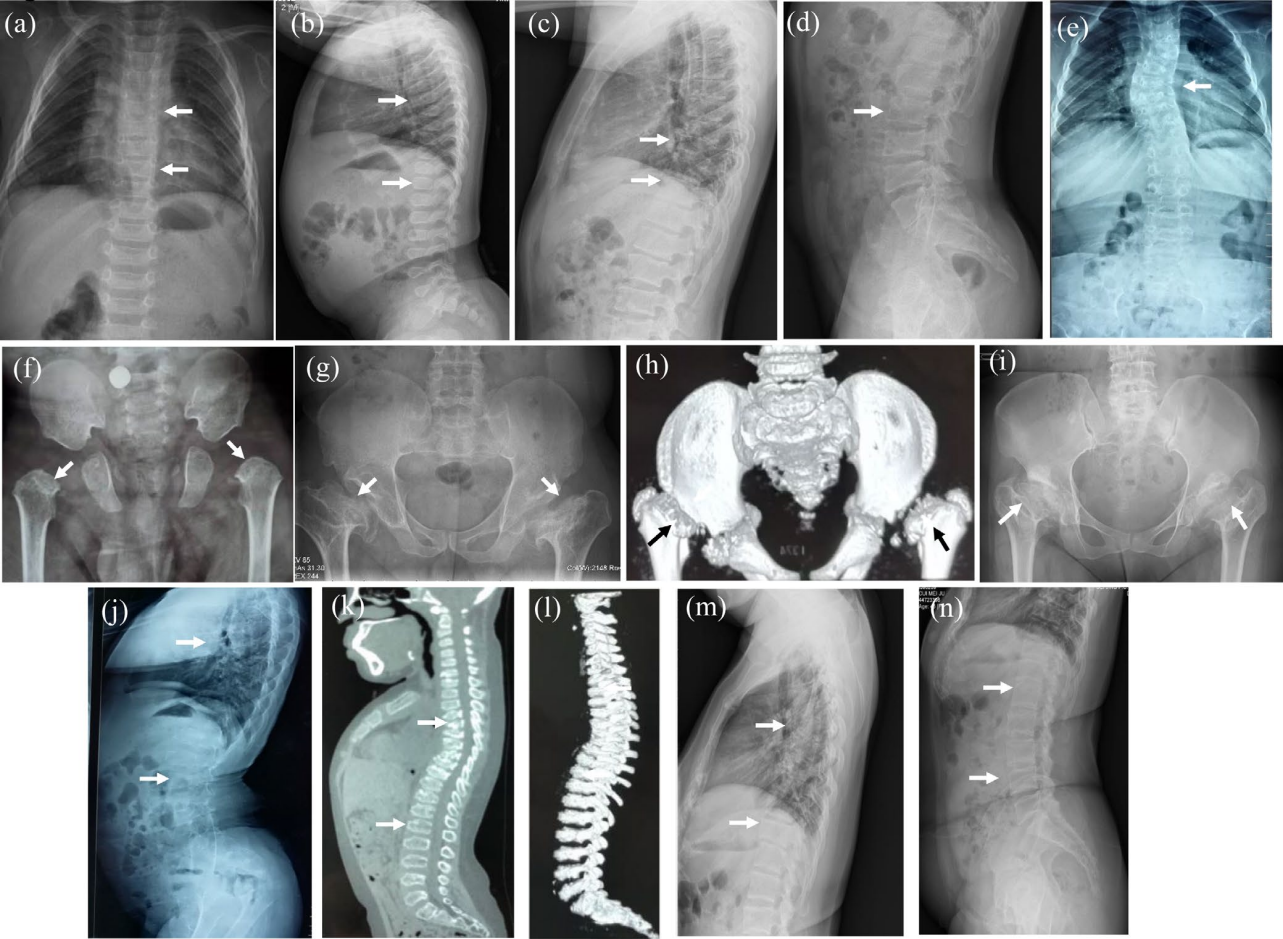
Imaging features of four spondyloepiphyseal dysplasia congenita patients with *COL2A1* variants. Proband 1. X‐ray films revealed flattened vertebral bodies, kyphosis, lumbar lordosis (a, b), flattened acetabular roof, compressed femoral heads, femoral heads epiphyses dysplasia and shortened femoral necks (f). Proband 1's mother. Radiographs revealed mild kyphosis, lumbar lordosis (c, d), flattened acetabular roof, and shortened femoral necks (g). Proband 2. Radiographs showed flattened vertebral bodies, kyphosis, lumbar lordosis and scoliosis (e, j–l), flattened acetabular roof, compressed femoral heads, femoral heads epiphyses dysplasia, and shortened femoral necks (h). Proband 2' father. X‐ray films revealed mild kyphosis, lumbar lordosis (m, n), flattened acetabular roof, and shortened femoral necks (i)

Proband 1 (II:1 of family 1) was a 2‐year‐old boy who came from a nonconsanguineous family. He was delivered by full‐term cesarean section with a birth weight of 3,450 g and height of 48 cm (*Z*‐score: −2.4). Growth retardation became obvious and progressive since 5 months of age, and he felt pain in the hip, especially after long‐distance walking. Physical examination revealed short‐trunk dwarfism with a height of 78 cm (*Z*‐score: −2.9), large head, short neck, shortening of the long arms, lumbar lordosis and adduction deformity of hips. His intelligence, hearing and eyesight were normal. Serum levels of Ca, P, ALP and PTH were within age‐specific normal range. Serum β‐CTX level was 2.1 ng/ml, and we could not judge if it was normal because the normal range was unavailable in Chinese children of this age. Serum 25OHD level was 21.0 ng/ml. Radiographic examination revealed mild lumbar lordosis, flattened and irregular vertebral bodies, flattening of the acetabular roof and compressed femoral heads (Figure 2a,b,f). BMD at LS 2–4 and femoral neck were 0.329 g/cm^2^ (*Z*‐score: −3.4) and 0.486 g/cm^2^ (*Z*‐score: −1.6), respectively.

The father (I:1 of family 1) of the proband was also affected with SEDC. He was 33 years old, with a height of 150 cm (*Z*‐score: −3.8). His growth was markedly tardy and he had pain in the hip with a waddling gait since 5 years of age. Physical examination revealed a disproportionately shortened trunk, short neck, kyphosis, genu valgum, and eversion of feet. He had mild hearing impairment, but eyesight and intelligence were normal. X‐ray films indicated mild lumbar lordosis and platyspondyly, flattened acetabular roof and dysplasia of bilateral femoral heads (Figure 2c,d,g). The mother of proband 1 was 30 years old without short stature or skeletal deformities.

#### 3.1.2 Family 2

Proband 2 (II:2 of family 2), a 11‐year‐old girl, was the second child of nonconsanguineous family. She was born at full‐term cesarean section with a birth weight of 3,750 g. At 1 year, motor delay and waddling gait were noticed. At 9 years of age, she suffered from back and knee pain, and limited motion of elbows. Physical examination revealed a short neck and trunk, a barrel‐shaped chest, severe kyphosis, lumbar lordosis with limited mobility of spine, adduction deformity of hips, enlarged and valgus deformity of knees, and waddling gait. X‐ray films indicated severe kyphosis and lumbar lordosis, flattened and irregular vertebral bodies. The three‐dimensional CT scan of pelvis revealed flattening of the acetabular roof, epiphyseal dysplasia of the femoral heads and shortening of the femoral necks (Figure 2e,h,j–l). Laboratory data showed normal levels of serum Ca, P, ALP, and PTH. Serum β‐CTX level was 1.31 ng/ml and 25OHD level was 24.4 ng/ml. BMD at L2‐4 and femoral neck were 0.463 g/cm^2^ (*Z*‐score: −2.1) and 0.574 g/cm^2^ (*Z*‐score: −2.0), respectively.

Proband 2's mother (I:2 of family 2) was 44 years old with a short stature, short neck, a barrel‐shaped chest and waddling gait. Her growth was markedly delayed since 9 years of age, and she had hip pain for two years. She did not experience bone fracture. Radiographs showed mild kyphosis, lumbar lordosis, flattening of the acetabular roof and shortening of the femoral necks (Figure 2m,n,i). Her BMD was 1.103 g/cm^2^ (*Z*‐score: 0.0) at LS and 1.048 g/cm^2^ (*Z*‐score: 1.7) at femoral neck. The proband's father was 50 years old with a height of 160 cm, and the height of the proband's 22‐year‐old brother was 170 cm. Her father and brother had no bone deformities and bone pain.

### 3.2 Disease‐associated variants

**Figure 3:**
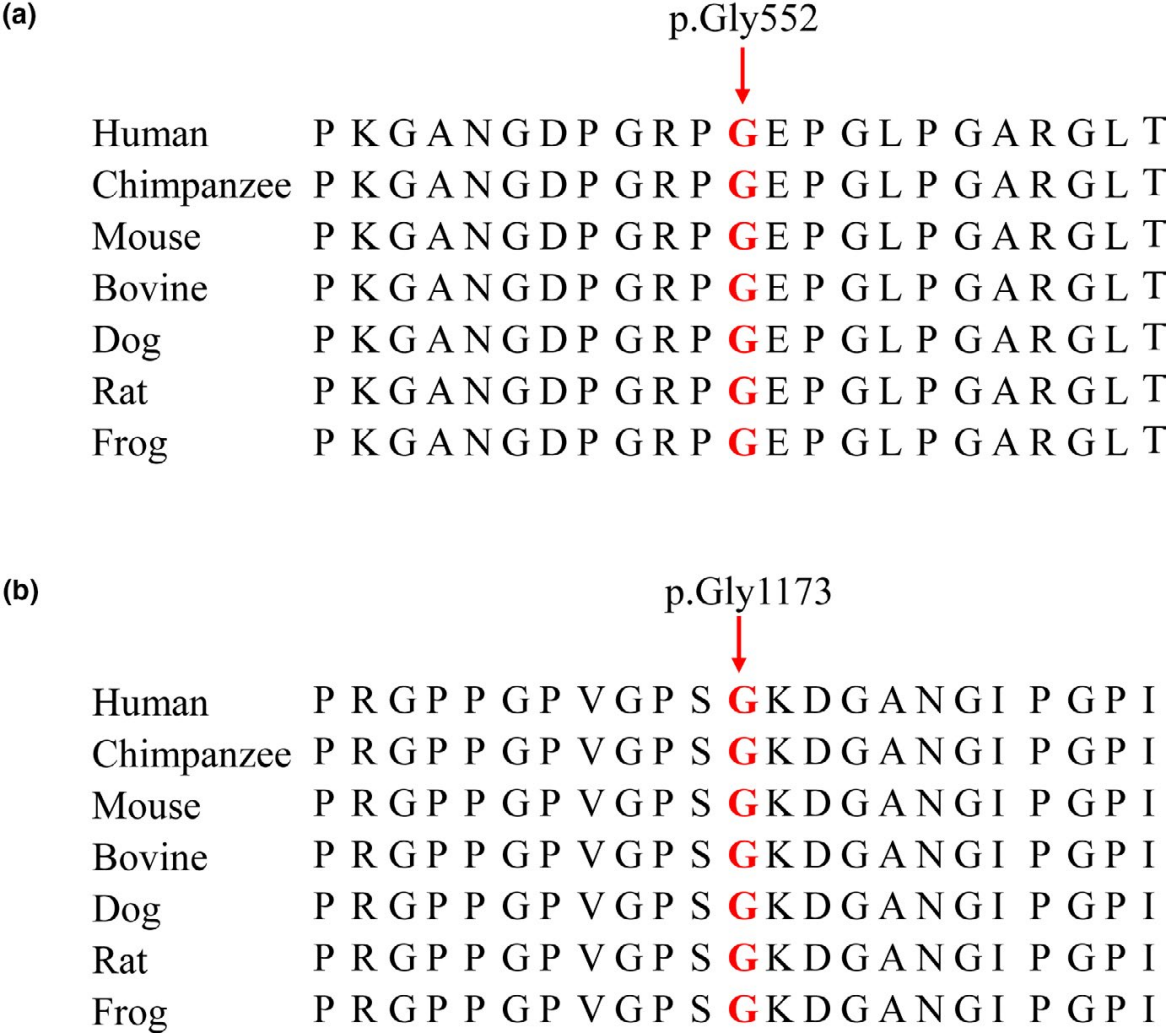
The conservatism of the amino acid substitutions among species. (a) p.Gly552 residue in type II collagen (NP_001835.3) was highly conserved among seven different species. (b) p.Gly1173 residue in type II collagen (NP_001835.3) was highly conserved among seven different species

Proband 1 and his father carried a missense variant of c.1654G>A in exon 25 of *COL2A1* (Figure 1c), which caused a substitution of glycine‐to‐arginine at codon 552 (p.Gly552Arg). A guanine‐to‐thymine substitution at position 3518 (c.3518G>T) in exon 50 of *COL2A1* was identified in the proband 2 and her mother, which resulted in an amino acid change of glycine‐to‐valine at position 1173 (p.Gly1173Val; Figure 1d). These variants in patients with SEDC were absent in the other family members and 100 unaffected controls. The variants had not been reported in various databases, including HGMD (http://www.hgmd.cf.ac.uk/ac/index.php), LOVD 3.0 (http://www.lovd.nl/3.0/home) and PubMed database. These two variants were predicted to be damaging by bioinformatics algorithms of PolyPhen‐2, SIFT and MutationTaster. Both variants were implicated as likely pathogenic using the ACMG guidelines. Moreover, the affected amino acid residues were highly conservative in type II collagen among different species (Figure 3a,b), indicating its functional importance throughout evolution.

## 4 DISCUSSION

Spondyloepiphyseal dysplasia congenita was an extremely rare genetic disorder, which was mainly caused by pathogenic variants in *COL2A1*. We identified two novel heterozygous variants of c.1654G>A (p.Gly552Arg) in exon 25 and c.3518G>T (p.Gly1173Val) in exon 50 of *COL2A1* in two unrelated Chinese families, which led to severe SEDC. The phenotypes of the probands included short‐trunk stature, flat face, short neck, barrel‐shaped chest and kyphosis, severe lumbar lordosis with limited mobility of spine and elbows, adduction deformity of hips, valgus deformity of knees, and waddling gait.

Human beings have a variety of collagen proteins, including collagen type I, II, IV, VI, IX, X, and XI. Type II collagen predominantly exists in the matrix and chondrocytes, including hyaline cartilage, the vitreous humor of eyes and the disk of the inner ears (Deng, Huang, & Yuan, 2016; Luo et al., 2017). Type II collagen was susceptible to be affected by *COL2A1* variants as its synthesis process was very complicated. The *COL2A1* variants could lead to many forms of chondrodysplasias and cartilage degeneration (Liu et al., 2018). The type II procollagen homotrimer was characterized by a triple‐helical configuration with three α‐1 chains folding together, of which 330 repeating Gly‐X‐Y triplets were included (Zabel et al., 1996). Amino acid substitution in the triple‐helical domain of procollagen α‐1 chains could slow down the folding of the protein into the triple‐helical conformation and impair the intracellular transport of collagen, thus leading to post‐translational overmodification of the protein and damage of the cartilage homeostasis and long bone development (Anderson et al., 1990; Godfrey & Hollister, 1988).

Spondyloepiphyseal dysplasia congenita could be induced by many kinds of variants in *COL2A1*, most of which resulted in structural changes within the triple‐helical domain of type II collagen (Nishimura et al., 2005; Xia et al., 2007). In this study, variants of c.1654G>A in exon 25 and c.3518G>T in exon 50 of *COL2A1* could induce amino acid substitutions (p.Gly552Arg and p.Gly1173Val) in the triple‐helical glycine residue, which were reported for the first time in *COL2A1*. p.Gly552Arg and p.Gly1173Val variants would damage the Gly‐X‐Y triplet sequence, which led to the degradation of premature collagen molecules, and led to abnormal bone and joints in patients with SEDC. Eighteen variants of *COL2A1* were reported to cause SEDC in Chinese including our patients, all of which were missense variants and affected the Gly‐X‐Y triple‐helical region (Cao et al., 2012; Chung et al., 2013; Huang et al., 2015; Li et al., 2014, 2015; Liu et al., 2016; Xiong et al., 2018; Xu et al., 2014; Zhang, He, Fu, Zhang, & Zhang, 2011). Moreover, variants in C‐terminal propeptide of type II collagen were described in other races, which led to atypical skeletal phenotypes (Kusano et al., 2017; Nishimura et al., 2005; Terhal et al., 2015).

We reviewed previously reported seven SEDC cases (three females, two males and two unknown sex) with glycine‐to‐valine substitution and six cases (two males and four unknown sex) with glycine‐to‐arginine to analyze the possible genotype–phenotype correlations. SEDC patients with p.Gly351Val and p.Gly396Val substitutions were reported with SEDC patients with p.Gly351Val and p.Gly396Val substitutions were reported with severe dens hypoplasia and myopia (Terhal et al., 2015), but they were not included in Table 2 due to lack of other clinical and radiological information. In patients with glycine‐to‐valine substitution, short‐trunk dwarfism was found in all SEDC patients, except patient 7. Hypoplasia of scoliosis (7/7), lumbar lordosis (7/7), femoral head dysplasia (6/7), platyspondyly (5/7), kyphosis (5/7) and, flat acetabular roof (4/7) were the most common symptoms of SEDC patients with glycine‐to‐valine substitution (Table 2). Short neck and genu valgum emerged in three patients (3/7), and none had brachydactyly. For extra‐skeletal features, two patients had cleft palate (2/7), and no patients had myopia, retinal detachment, nuclear cataract or hearing impairment (Table 2). Because clinical and radiological information were unavailable in patients with p.Gly687Arg, p.Gly831Arg, p.Gly813Arg and p.Gly1101Arg variants (Barat‐Houari, Dumont et al., 2016; Polla et al., 2015), only four patients with glycine‐to‐arginine substitution are included in Table 3. These patients showed similar skeletal phenotypes with glycine‐to‐valine substitution, but they were more likely to have extra‐skeletal impairment.

**Table 2:** Summary of SEDC patients with glycine‐to‐valine substitution

Patient no.	1	2	3	4	5	6	7
Ethnicity	Chinese	Chinese	Chinese	Chinese	Chinese	Korean	Caucasian
Gender	F	F	M	F	F	M	F
Age (year)	11	44	12	15	26	21	18
Height (cm)	110.5	146	124	115	115	125	153
Weight (kg)	31	58	28	NA	26	43	NA
Family history	+	+	−	−	+	−	−
Birth length (cm)	NA	NA	NA	NA	NA	NA	NA
Birth weight (g)	3,750	NA	NA	NA	NA	3.5	NA
Onset	1 years	9 years	At birth	At birth	At birth	5 years	13 years
Short trunk	+	+	+	+	+	+	−
Short neck	+	+	−	−	−	+	−
Cleft palate	−	−	+	−	−	+	−
Scoliosis	+	+	+	+	+	+	+
Kyphosis	+	+	+	+	−	‐	+
Lumbar lordosis	+	+	+	+	+	+	+
Platyspondyly	+	−	+	+	−	+	+
Flat acetabular roof	+	−	+	+	−	+	−
Femoral head dysplasia	+	−	+	+	+	+	+
Genu valgum	+	−	−	+	−	−	−
Feet deformity	−	−	−	+	−	+	−
Brachydactyly	−	−	−	−	−	−	−
Osteoporosis	+	NA	+	NA	+	NA	NA
Myopia	−	−	−	−	−	−	−
Retinal detachment	−	−	−	−	−	−	−
Vitreoretinal degeneration	−	−	−	−	−	−	−
Nuclear cataract	−	−	−	−	−	−	−
Hearing impairment	−	−	−	−	−	−	−
Variant	p.Gly1173Val	p.Gly1173Val	p.Gly1176Val	p.Gly1149Val	p.Gly1086Val	p.Gly277Val	p.Gly204Val
Literature	This study	This study	Cao et al. (2012)	Liu et al. (2016)	Li et al. (2014)	Jung et al. (2004)	Rukavina et al. (2014)

Abbreviations: +, phenotype was dominant; −, phenotype was absent; Gly, glycine; NA, not available; SEDC, spondyloepiphyseal dysplasia congenita; Val, valine.

**Table 3:** Summary of SEDC patients with glycine‐to‐arginine substitution

Patient no.	1	2	3	4
Ethnicity	Chinese	Chinese	European	European
Gender	M	M	M	M
Age (year)	2	33	16	7
Height (cm)	78	150	NA	87
Weight (kg)	12.5	55	NA	NA
Family history	+	+	−	+
Birth length (cm)	48	NA	49	44
Birth weight (g)	3,450	NA	4,450	3,220
Onset	5 months	5 years	4 months	2 months
Short trunk	+	+	+	+
Short neck	+	+	+	+
Cleft palate	−	−	−	−
Scoliosis	+	+	NA	+
Kyphosis	+	+	NA	−
Lumbar lordosis	+	+	+	+
Platyspondyly	+	−	+	+
Flat acetabular roof	+	+	+	+
Femoral head dysplasia	+	+	+	+
Genu valgum	+	+	−	−
Feet deformity	−	−	−	−
Brachydactyly	−	−	−	−
Osteoporosis	+	NA	NA	NA
Myopia	−	−	−	+
Retinal detachment	−	−	−	+
Vitreoretinal degeneration	−	−	−	−
Nuclear cataract	−	−	−	−
Hearing impairment	−	+	−	+
Variant	p.Gly552Arg	p.Gly552Arg	p.Gly154Arg	p.Gly973Arg
Literature	This study	This study	Vikkula et al. (1993)	Sobetzko et al. (2000)

Abbreviations: +, Phenotype was dominant; −, phenotype was absent; Arg, arginine; Gly, glycine; NA, not available; SEDC, spondyloepiphyseal dysplasia congenita.

The phenotypic spectrum of SEDC widely varied, and the same variant in a family could produce diverse phenotypes. We found that patients with p.Gly1176Val, p.Gly1173Val and p.Gly1149Val variants had relatively more severe skeletal deformities than those with p.Gly1086Val, p.Gly277Val and p.Gly204Val substitutions, which was consistent with a recent study (Liu et al., 2016). They demonstrated that variants in exons 49–50 could lead to more severe spinal deformities and hip dysplasia, and more extra‐skeletal anomalies such as cleft palate (p.Gly1176Val) and hearing impairment (p.Gly1152Asp; Liu et al., 2016). However, the probands with glycine‐to‐valine substitution rarely suffered from extra‐skeletal abnormalities, while patients with glycine‐to‐nonvaline and other substitutions could get nuclear cataract, myopia, hearing loss and so on (Chung et al., 2013; Liu et al., 2016; Xu et al., 2014; Zhang et al., 2011). In our study, patients with same amino acid substitution had different severity of phenotypes and extra‐skeletal abnormalities, which suggested that genetic and environmental factors could have impact on phenotypes of SEDC. At present, no effective medications or molecular targeted therapy are available for SEDC. For severe skeletal deformities, surgical correction could be useful to relieve pain, improve movement, and to delay the progressive thoracolumbar scoliosis (Bayhan et al., 2017; Serhan Er et al., 2017).

Our study had several limitations. First, we did not assess the effects of the variants on type II collagen, so the underlying mechanism of *COL2A1* gene variants inducing SEDC remained unclear. Second, cartilage biopsy was not done because it was an invasive procedure. Last, it was difficult to establish a certain genotype–phenotype correlation because of the small sample of this study.

In conclusion, we identified two novel heterozygous variants of c.1654G>A (p.Gly552Arg) and c.3518G>T (p.Gly1173Val) in *COL2A1* in two unrelated Chinese families with severe SEDC. The variants might induce SEDC by impairing type II collagen triple helix formation and leading to damage of the cartilage homeostasis. Our findings expanded the genotypic and phenotypic spectrum of extremely rare type II collagenopathies.

## CONFLICT OF INTEREST

The authors have no conflict of interest.

## AUTHOR CONTRIBUTIONS

W.B.Z. carried out the molecular genetic studies, participated in the sequence alignment, analyzed data, and wrote the manuscript. L.J.L. and D.C.Z. contributed to data collection. O.W., Y.J., X.P.X., and W.B.X. reviewed the manuscript. M.L. contributed to the conception and design of the research, acquisition and interpretation of the data, and revised the manuscript.

## Data Availability

The data files used to support the findings of this study are available from the corresponding author upon request.
